# Supplementary material for: MetaRibo-Seq measures translation in microbiomes
Source: Nat Commun. 2020 Jun 29;11:3268. doi: 10.1038/s41467-020-17081-z (PMC7324362; doi:10.1038/s41467-020-17081-z)
Supplement: Supplementary file 10 — Supplementary Data 7 [file 41467_2020_17081_MOESM10_ESM.zip › File2/Confidence_High_Taxonomy/4642_out.krona.html]

Javascript must be enabled to view this page.

members
magnitude
magnitudeUnassigned
count
unassigned
taxon
rank

4642\_out

3

3
superkingdom
2

1239
phylum
3

186801
class
3

186802
order
3

3
family
186803

841
genus
3

species

SRS014923\_contig\_number\_31511SRS015431\_contig\_number\_48076SRS148721\_contig\_number\_14629
3
1262943
